# Selections

**Published:** 1892-12

**Authors:** 


### *SELECTIONS.*

---

### The Principles of Treatment in Gonorrhœa.

At the late meeting of the International Dermatological Con gress, Prof. Neisser, of Breslau, formulated the rules for the treatment of gonorrhœa in the following conclusions :

I. The basis of all prophylactic and therapeutic measures in this disease is the recognition of the gonococcus as the cause of gonorrhœal infection. It is necessary in every stage of the affection to determine the presence and seat of the lesion. The diagnosis is impossible in many acute cases and in all subacute and chronic cases without microscopical examination of the secretion for gonococci; microscopic inspection alone, especially in women, is perfectly worthless.

II. The dangers of gonorrhœa are :

1st. That the gonorhœal virus and the lesions resulting therefrom may not remain confined to the mucous area originally infected. In men the posterior urethra—so inaccessible to therapeutic measures—the seminal ducts and epididymis may be attacked, and prostatic and vesical complications may arise. In females the uterus, tubes, ovaries and peritoneum may be affected.

2d. That during the latter stages the gonorrhœal virus extends to the deeper epithelial layers.

Owing to this extension in both directions, both over the surface as well as into the deeper parts, it comes about that the virus may remain for months or years in places difficult of access or inaccessible ; that is may form a chronic source of infection. It is only during the first stages that the virus is found in accessible places (in males the anterior urethra, in females the urethra and cervix) and in the superficial layers of epithelium, so that the disease is easy to treat, even by the patient himself.

The aim of treatment in gonorrhœa should, therefore, be to prevent conversions of an anterior into a posterior urethritis and of an acute into a chronic urethritis. It should therefore be commenced as early as possible after infection.

III. Only medicaments should be employed which *a.* destroy gonococci ; *b.* increase the inflammation as little as possible ; *c.* have no injurious action upon the mucous membrane, such as solutions of nitrate of silver 1-4000 to 1-2000, ammonia sulpho-ichthyolate 1-100, sublimate 1-30,000 to 1-20,000.

Improper are remedies which are simply astringent on account of the danger of transporting the virus by the injection.

---

### PRACTICAL HINTS REGARDING CHILDREN.

Always teach a nurse that a child cannot swallow as long as the spoon is between the teeth; that it is advisable to depress the tongue a brief moment, and withdraw the spoon at once, and now and then a momentary compression of the nose is a good adjuvant.

The taste of quinine is disguised by coffee, chocolate and "elixir simplex."

Powders must be thoroughly moistened; unless they be so the powder adhering to the fauces is apt to produce vomiting.

Inunctions require a clean surface, and are best made where epidermis is thin, and the lymph-ducts very extensive, as on the inner aspect of the forearm and the thigh.

Babies, after having taken opiates for sometime, demand larger, and sometimes quite large, doses to yield a sufficient effect.

Febrifuges and cardiac tonics, such as quinine, antipyrine, digitalis, strophanthus, sparteine, convallaria, etc., are tolerated and demanded by infants and children in larger doses than the ages of the patients would appear to justify.

Mercurials affect the gums very much less in young than in advanced age.

The rectum of the young is straight, the sacrum but little concave, the sphincter ani feeble, and self-control is developed but gradually; for these reasons, rectal injection is allowed to flow out or is vehementally expelled. Therefore one which is expected to be retained must not irritate. The blandest and mildest is a solution of six or seven parts of chloride of sodium in a thousand parts of water, which serves as a good vehicle for medicine unless incompatible with the latter. The injection must be made while the child is lying on its side (preferably the left side), not on the belly over the lap of the nurse, for in this position the space inside the narrow infantile pelvis is reduced to nothing.

In many cases of intense intestinal catarrh, large and hot (104° to 108° F.) enemata will relieve the irritability of the bowels and contribute to recovery. They must be repeated several times daily. When there are many stools, and these complicated with tenesmus, an injection, tepid or hot, must or may be made after every defecation, and will speedily relieve tenesmus.—*Prof. Jacobi, Pacific Med. Journal.*

---

### MANAGEMENT OF NORMAL LABOR.

Prof. Schauta, of Prague, says (*Dietetic Gazette*):

The chief elements in the treatment of normal labor may be summed up concisely in the two words, "cleanliness and rest."

With regard to cleanliness, this should be insisted on before the confinement. The woman must, therefore, make her genitals aseptic before labor and keep them so afterwards.

The abdomen, hips, thighs, inguinal folds, etc., should be washed with soap and sublimate 1-1000. Then the vagina should be rinsed with 2 per cent. carbolic solution, while the fingers distend the vagina. The physician's hands must be most scrupulously aseptic; as also the instruments and bandages which may be used, made so by hot air or boiling.

Immediately after the delivery, the genitals are to be carefully wiped off with warm cabolic solution. Ordinarily, the vagina is not washed. Indications for the same are birth of a foul fœtus, discolored amniotic fluid and operative cases.

The cleansing after labor is therefore confined to the external genitals. It consists of cleansing with carbolic solution two or three times a day. Wounds, be they ever so small, must be closed by suture immediately after birth and covered with aseptic cotton, which is to be renewed at least three times a day.

Further treatment must consist in attention to the abdomen, breasts, bladder and the rectum. If ischuria is present, it is treated by laying cotton, which has been dipped in hot water, upon the external genitals. If this does not help, the catheter is to be carefully introduced. The vicinity of the meatus should be carefully cleansed, and the instrument is only to be used after having been boiled for at least ten minutes, and *catheterization should not be done under the bed clothes.*

If, after three days there has been no passage, then rectal irrigation (1-2 quarts) must be practiced. With women who wish to nurse their own children, the treatment of the nipples must commence before confinement, by drawing them forward and bathing them with alcohol. If the breasts of non-nursing women are swollen, then a light compression by means of bandages over the breasts and shoulders, in the form of the spica, recommends itself.

---

### POINTS TO OBSERVE IN EXCISION OF THE BREAST.

Two considerations only should guide the surgeon. The first is imperative; it refers to the complete removal of the nipple and skin over the tumor, when that is malignant. The second is to have the cicatrix parallel to the fibers of pectoralis major. In most cases both of these objects are best attained by the same incision—one inclosing an ellipse of skin parallel with the anterior fold of the axilla when the arm is at a right angle with the trunk. It is stated that it is sometimes convenient to prolong the incision into the axilla, but never necessary, as the axilla can be readily reached from any incision from amputation of the breast. In cases of carcinoma, the glands should be removed from the axilla, together with the mass of fat in which they are situated. The intercosto-humeral nerve should be preserved. Bleeding vessels are caught with pressure-forceps as fast as they are cut. Occasionally one or more arteries will require twisting. A ligature is never necessary. The wound is to be thoroughly flushed with bichloride solution 1-2000. A continuous suture of fine chromicized catgut is recommended, each loop being caught up —the buttonhole stitch. A drainage tube is not to be employed. The dressing should be aseptic and it should secure accurate apposition of the wound surfaces. The author uses four layers of boric lint to lay over the wound, the margins extending an inch beyond the wound in all directions. This is held in place by strips of plaster two inches wide. Over this gauze or wool is applied, and held in place by a roller bandage. The arm is held to the side by means of an ordinary chamber towel. The towel is folded lengthwise, and between the two folds the forearm and arm are placed, the hand being just within one end. The towel is then fixed in place by pins. After the first day the patient may be raised to a sitting position by pillows or a bed-rest. The dressing may be removed on the seventh day, when the stitches may be taken out. The wound is re-dressed by two layers of sublimate gauze, fixed with collodion, and over this a light boric lint dressing held with the roller bandage.—*London Lancet.*—*Pacific Med. Jour.*

---

### PRESENT STATUS OF SURGERY OF THE GALL BLADDER.

Czerny, in the *Deutsche Medicinische Wochenschrift*, reviews the history of the surgery of the gall bladder and discusses the indications for operation. He divides the cases into those in which marked jaundice is wanting and those in which it is present, and briefly details three cases in which he performed cholecysto-enterostomy. The following is his summary :

(1) Gall-stones call for operative measures when they give rise to repeated and long-lasting troubles.

(2) Empyema always calls for operation and also hydrops, when it gives rise to serious symptoms.

(3) The typical operation for stone in the gall bladder consists in incision, emptying and suturing of the bladder, the abdominal wound being drained for a short time.

(4) When the cystic duct is occluded, or the gall bladder itself is inflamed, or when its contents are much altered, a temporary fistula of the gall bladder should be made.

(5) Extirpation of the gall bladder is only indicated by markedly inflammatory or carcinomatous conditions.

(6) In occlusion of the ductus choledochus operation is always indicated when the strength of the patient permits. When it is not possible to remove the obstruction (stone or bending), a fistula should be established between the gall bladder and the duodenum.

(7) Access is best obtained in operations on the gall bladder by means of an L-shaped incision, the longitudinal branch of which is made in the linea alba and the horizontal to the right (patient's) from a point immediately below the umbilicus.

(8) The danger to life of operations for gall stones appears to be less than in that for the removal of stone in the urinary bladder.—*Univ. Med. Mag.*—*Pacific Med. Jour.*

---

### THE MOST SUCCESSFUL TREATMENT OF HYPERTROPHY OF THE TONSILS.

Dr. Th. Goureau writes as follows (*Merck's Bulletin*): Much has recently been written concerning the best treatment to be adopted in hypertrophy of the tonsils, and here, as elsewhere, opinions are much divided. All the processes may, in short, be referred to three principal methods: painting, excision, the actual cautery.

After carefully comparing the three methods, we may state that the preferable process is excision by means of Maisonneuve's Amygdalotome. This instrument, which is in the hands of every practitioner, is certainly the one which gives most satisfaction with the least drawbacks. We do not say that it must always be employed; it is foolish to be absolute in practice. But eight times out of ten recourse must be had to amygdalotomy.

What are, then, the contraindications?—1st, certain anatomical conditions of the tonsils; 2d, the age of the subject. It may not be possible to seize the tonsil by the instrument; its too friable tissue being torn by the fourchette; or the pillars covering the tonsils, adherent to them, prevent them from being caught in the lunette. In the case of adults, amydalotomy should be forbidden. The sole reason for this direction is the possibility of fatal hemorrhage; for after infancy the tonsilary vessels are developed; their compression is difficult; their ligature impossible. The hemorrhage often comes in the form of venous exudation.

At what age does this contraindication begin? The author replies: "Before puberty, amygdalotomy may be practiced, after,—after, never." Double amygdalotomy ought to be avoided; and it is necessary that the surgeon have in his instrument-case simple amygdalotomes of different sizes; for the tonsil ought to enter the lunette readily, but it should not be loose in it.—*Ex.*

---

### SOME PRACTICAL POINTS IN THERAPEUTICS.

Dr. Jno. A. Larrabee, in a paper recently published (*Med. and Surg. Rep.*) made mention of those therapeutic agents which, during the summer months, had been so successful, in his hands. In entero-colitic diarrhœa—the so-called "summer complaint" of cities, dependent upon the various micro-organisms, vitiated air and bad food—salol, naphthaline, carbolic acid, calomel, in minute doses, and nitrate of silver stood the test.

In gastro-enteritis he found salicylate of bismuth useful, and in inflammatory diarrhœas of infants and older children Rochelle and Epsom salts, in acid infusion of roses with small doses of laudanum. In chronic cases the nitrous acid camphor mixture (Hope's) did not fail.

He also used this treatment very satisfactorily in the exanthemata.

In diphtheria peroxide of hydrogen and whiskey internally gave him the best results. Disappointment in the use of the peroxide is often due to the poor quality of the drug. It is a very unstable article. It should always be purchased in small amounts protected from the light, by being put in 4 oz. bottles.

On coming in contact with the false membrane of the throat, a white foamy reaction takes place. It should be freely applied to the throat on mops or by use of spray. The crying and resistance of the child, favors the distribution of the fluid. In this way the author has treated successfully a great many cases of diphtheria. He believes that the symptomatic use of definite, although often toxical, doses of whisky, in children even of tender age, to be the surest safeguard against heart failure.—*Ex.*

---

### THE LATEST ANALYSIS OF THE KEELEY CURE.

Hydrochlorate of apomorphine is said to be the agent responsible for all the ill effects of the Keeley "bichloride of gold" cure. This we have on the authority of one who has compounded large amounts of the whisky employed as an adjuvant to the cure. The feeling of nausea and sickness which follows each dose of the whisky as supplied by the Keeley-cure people is ascribed usually to the action of the atropine and strychnine contained in the fluid employed for hypodermatic injection, and no one has ever suspected that the fits of trembling, symptoms of paralysis, and general collapse which develop in so many of the "victims," are due to aught but the effects of the injected fluid, or the natural consequence of the withdrawal of alcoholic stimulants from a person whose system craves the stimulating effects of alcohol. To illustrate the dangerous nature of this drug, we quote Hare, who says that Reichert found that apomorphine hydrochlorate in poisonous doses produces convulsions and finally paralysis which is spinal in its origin. "On the nervous centers in the brain apomorphine acts as a stimulant. . . . The motor and sensory nerves are finally paralyzed, and even the muscles become poisoned and incapable of contraction."

Each ordinary dose of the whisky is said to contain $\frac{1}{50}$ grain of apomorphine ; and, as $\frac{1}{10}$ grain of the drug is necessary to produce emesis, it can be easily understood in what manner the decoction acts.—*Pharmaceutical Record.*

### THE VALUE OF LITHIA WATERS.

It is one of the curious developments of modern medicine that remedies largely used by practitioners for years are suddenly shown to be lacking in the powers generally attributed to them. For years the profession has used lithia water in various diseases with the idea that the results obtained were due to the comparatively small quantity of lithia present in solution. Those physicians who examined the subject closely speedily concluded that the greater part of the benefit derived by patients from so-called lithia waters depended rather on the large amount of pure water ingested than upon the lithia contained in it. In other words, the pure water practically flushed the body of impurities. These conclusions were still further supported by the discovery on analysis that one of the widely-advertised lithia waters, indorsed by a large number of misguided persons, was only a pure water, with practically not a trace of lithia in it. Still more recently Haig has told us that while lithia speedily combines with uric acid in a test-tube, in the body it has a greater affinity for the acid sodium phosphate in the blood, and combining with this leaves the uric acid untouched. Lithia waters should be used not for their lithia but for their purity, and the results obtained placed to the credit of the flushing of the system, not to the lithia. A recent advertisement by one of the lithia water companies states that one of the editors of the *Therapeutic Gazette* strongly recommends lithia water, and not only this, but states that their particular brand is the best. The truth is that he has never employed their commodity, and has only recommended another brand of lithia water when *no other* pure water is obtainable.—*Therapeutic Gazette.*

---

### OBJECTIVE SIGNS OF NEURASTHENIA.

In the *Munich med. Woch.*, Loewenfield advises that all cases of neurasthenia be examined for the following signs ; General nutrition remains unimpaired unless there is dyspepsia. The skin of the face and chest reddens at the least emotion, the ear and cheek sometimes being deeply injected. Narrowing of the visual field is exceptional. There is excessive mobility of the pupils, that grow smaller and larger not only on account of ordinary physiological excitation, but from psychic impressions as well. Inequality of the pupils occasionally present is transitory. Muscular phenomena are complex. There is inability to close the lids completely, weakness of movement in convergence, motor insufficiency in hands and arms, and fibrillary twitchings. Reflexes are often exaggerated. Loss of knee-jerk is not a symptom of pure neurasthenia. Nervous excitability is exaggerated, especially that of the optic nerve. Troubles with the voice, speech and writing are due to lessened resistance of the muscles to fatigue. The heart-beats are either increased or diminished. Vascular tone is lessened, and the arteries are lengthened in serpentine form. The motor force of the stomach is diminished. and there is more or less hyperacidity. Eructations, constipation and nervous diarrhœa complete the dyspeptic symptoms. General hyperidrosis is common, though sweating may be confined to the hands and feet, or to the head during sleep. The urine nearly always undergoes some change. There may be mucus in it; polyuria, phosphaturia, or oxaluria may exist.—*Medical Record.*

---

### HEADACHE DUE TO TRAVEL ON RAILROAD TRAINS.

Dr. A. N. Blodgett (Boston Medical and Surgical Journal), in discussing the subject of ocular headaches, referred to a form of headaches resulting from travel on railroad trains, which he thought was more frequent than generally supposed. Treatment by any of the methods usually employed is generally without benefit. An explanation was once given him by Mr. Fox, the consulting engineer intrusted with the construction of the railway tunnel beneath the river Mersey at Liverpool. In the journey between Liverpool and London, Mr. Fox incidentally made the remark that he always sat with his back toward the engine. The English cars are built with transverse compartments, so that the passenger is obliged to sit on a fixed seat, and therefore half the persons in a compartment are forced to sit with their back toward the engine. Mr. Fox stated that he always took that position from the fact that his eyes were rendered much more comfortable during the journey. He thought that was due to the avoidance of the repeated and sudden strain in the accommodation which is rendered necessary if one is looking at a series of rapidly approaching objects, as when traveling in the train. The effect was like a blow upon the eye. If the traveler be looking backward, the objects would be constantly receding and the strain of accommodation was continually letting up, and caused no discomfort whatever. That seemed to be a very ingenious and logical explanation why some people suffer from headache and vertigo in railroad traveling. Since that time he has directed car-sick travelers to ride backward, and has adopted this method himself with the greatest comfort.—*Cincinnati Lancet-Clinic.*

---

### SALICYLIC ACID IN THE TREATMENT OF CHANCROIDAL BUBO.

Dr. Frederick W. Gwyer presented a patient to the New York Surgical Society upon whom he had practiced this treatment: The patient, a man aged twenty-two years, had entered Bellevue Hospital on April 20th. He had had connection five days before the appearance of a chancroid on the penis. Five days later a bubo had developed in the left inguinal region. At the time he entered the hospital an inflamed, fluctuating chancroidal bubo of the size of a hen's egg was found. Crystals of salicylic acid were applied to the sore on the penis. In a few days the active symptoms of the bubo had subsided and the tumor had diminished in size. Shortly afterward the tumor entirely disappeared and now not a sign of any trouble existed. The speaker thought the acid was absorbed by the lymphatics and carried to the enlarged glans. By this method of treatment objectionable scars were avoided. He had treated many other cases in the same way with equally good results.—*Ex.*

### STRYCHNINE FOR SHOCK, ETC.

Dr. H. A. Hare, before the Mississippi Valley Medical Association said (*Lancet Clinic*): I wish to call your attention to the use of strychnine as a remedy for and preventive of surgical shock and anæsthetic collapse, not to speak of its value in opium-poisoning. In these conditions atropine, while very useful, so far as its vaso-motor effects are concerned, does not compare with strychnine, either theoretically or practically. To those who habitually employ atropine and morphine injections prior to the use of an anæsthetic, let me recommend the use of strychnine or strychnine and atropine combined. There is one point to be remembered in regard to the use of strychnine in shock or accident, and that is to give it in full doses or leave it alone. Not less than $\frac{1}{20}$ grain should be employed hypodermically every half-hour in an adult, and, if the condition of shock or respiratory and cardiac failure be marked, one dose of as much as one-fifth grain may be given in this way. Disagreeable effects rarely, if ever, follow, and if they do, will amount to little more than muscular twitching, which can readily be governed by sedatives, for if the drug can stimulate the nervous system sufficiently to cause irritability, it will have pulled the patient out of the "Slough of Despond," and he will be able to stand further treatment should the effect of the strychnine be excessive. Under the conditions spoken of, the man is on the brink of death, and we cannot afford to make haste slowly in dragging him back.

---

### HOW LONG SHOULD A DIPHTHERIA CASE BE ISOLATED?

This most important question is discussed by Dr. Prince, in the *Boston Medical and Surgical Journal.* It is a matter of vital importance, for Dr. Prince tells us of a case wherein the patient, supposed to be well, made a visit to relations in Boston nine days from the date of his "getting up." One week after his arrival, a child in the family was attacked with diphtheria and died. An outbreak of diphtheria, in a hotel at Nantucket, followed the arrival of a person just recovering from diphtheria, and pronounced well by the attending physician. One of these cases, when supposed to be well, carried it to a hotel in town. Three cases of diphtheria in one family closely followed the advent of a nurse who had just come from attendance on a fatal case.

Dr. Prince thinks that evidence goes to show that poison is retained in the mucous membrane longer than is generally considered to be the case. In lieu of definite knowledge he has adopted the arbitrary rule of advising quarantine precautions for one week after the patient appears to be perfectly free from disease. This seems to be a fairly safe rule and one that is desirable.—*Annals of Hygiene.*

---

### ON THE TORSION OF ARTERIES.

In connection with operations for excision of tumors, and other excisions of a like character, Jonathan Hutchinson remarks as follows: "I may mention that for many years I have quite ceased to use any other means for arrest of arterial bleeding than torsion. In excision of the breast, for instance, I do not think that I have during the last fifteen years ever used a ligature. The torsion is always effected by a pair of Well's clamp-forceps, now in such universal employment. I am always extremely careful to close all vessels, keeping the wound exposed for a considerable time for that purpose. Very seldom indeed have I encountered any secondary hemorrhage."—*Columbus Medical Journal.*

---

### DIPHTHERIA AND ITS TREATMENT.

This treatment has been successful in thirty-seven consecutive cases. It consists in the application to the patches of a strong solution of argentic nitrate, gr. xx to ℥ss of rose water, by means of a throat brush. It must be done thoroughly and twice a day.

This procedure should be immediately followed by a gargle, *e. g.* :

℞. Tr. Kino, f ℥ii.

Glycerine, f ʒii.

Ol. eucalyptol, gtt. x.

S. : A teaspoonful in a tablespoonful of water as a gargle.

After this the throat should be dusted with the following:

℞. Hydrarg. chlor. corros., gr. i.
Pulv. sulphuris, ʒi.

S. : Blow a pinch into the throat every four hours.

Internally, the following mixture should be given according to the age of the patient:

℞. Pulv. potas. chlorat., iii.

W. ferri. chlor., f ii.

Syr. limonis, f ʒiii.

Ol. gaultheriæ, gtt. iii.

S. : A teaspoonful every two or three hours.

Apply externally over the site of the tonsil :

℞. Tr. iodi, f ℥ii.
Ol. camphorat., f ℥i.

S. : Apply every four hours.

When there is a rise of temperature give small doses of antipyrine. During convalescence use mist. ferri et ammon. acetatis, light nutritious diet and champagne.—*Dr. Alexander Fulton, Medical News.*

---

### THE TREATMENT OF ACUTE TONSILITIS.

Dr. Ch. Eloy, believing that acute tonsilitis is of microbian origin, contagious and as well infectious, strongly insists upon disinfection of the mouth, destruction of enlarged tonsils by ignipuncture to close the avenues of infection, and the segregation of those predisposed to this disease. Isolation, then, is social prophylaxis. He practices internal antisepis by salol, salicylates, naphthol, to prevent infection, or, if it has already occurred, to combat it. The diet is regulated, tonics, and antipyretics are ordered, not only to modify the symptoms but as well to place the organism in the best possible condition of defence against the invading microbes. —*Revue Gen. de Clin. et de Ther.* (*American Journal of the Medical Sciences*).

### MALARIAL CACHEXIA.

In a case of a man twenty-five years of age, with the Malarial Cachexia, Prof. Da Costa prescribed the following:

℞. Liquor. arsenici chloridi, gtt. v.
Tincture ferri chloridi, gtt. xx.
Elixir.
Aquæ, aa, q. s., ad, fluid dram j.

M. To be take three times a day, after meals.

Also, for the first four days, give quinine, gr. viij., in the morning on an empty stomach, and repeat the dose in the evening. Then continue by giving gr. viij. in the morning only; the quinine to be given in capsules or in powder.—*Medical World.*

